# Interim 2019/20 influenza vaccine effectiveness: six European studies, September 2019 to January 2020

**DOI:** 10.2807/1560-7917.ES.2020.25.10.2000153

**Published:** 2020-03-12

**Authors:** Angela Rose, Esther Kissling, Hanne-Dorthe Emborg, Amparo Larrauri, Jim McMenamin, Francisco Pozo, Ramona Trebbien, Clara Mazagatos, Heather Whitaker, Marta Valenciano, European IVE group

**Affiliations:** 1 Epiconcept, Paris, France; 2 Authors contributed equally to the study and manuscript writing; 3 Department of Infectious Disease Epidemiology and Prevention, Statens Serum Institut, Copenhagen, Denmark; 4 National Epidemiology Centre, Institute of Health Carlos III, Madrid, Spain; CIBER de Epidemiología y Salud Pública (CIBERESP), Institute of Health Carlos III, Madrid, Spain; 5 Health Protection Scotland, Glasgow, United Kingdom; 6 National Centre for Microbiology, National Influenza Reference Laboratory, WHO-National Influenza Centre, Institute of Health Carlos III, Madrid, Spain; CIBER de Epidemiología y Salud Pública (CIBERESP), Institute of Health Carlos III, Madrid, Spain; 7 Department of Virus and Microbiological Special diagnostics, National Influenza Center, Statens Serum Institut, Copenhagen, Denmark; 8 Public Health England, London, United Kingdom; 9 European Influenza Vaccine Effectiveness (IVE) group members are listed at the end of the article.

**Keywords:** influenza, vaccine effectiveness, multicentre study, test-negative design, Europe

## Abstract

**Background:**

Influenza A(H1N1)pdm09, A(H3N2) and B viruses were co-circulating in Europe between September 2019 and January 2020.

**Aim:**

To provide interim 2019/20 influenza vaccine effectiveness (VE) estimates from six European studies, covering 10 countries and both primary care and hospital settings.

**Methods:**

All studies used the test-negative design, although there were some differences in other study characteristics, e.g. patient selection, data sources, case definitions and included age groups. Overall and influenza (sub)type-specific VE was estimated for each study using logistic regression adjusted for potential confounders.

**Results:**

There were 31,537 patients recruited across the six studies, of which 5,300 (17%) were cases with 5,310 infections. Most of these (4,466; 84%) were influenza A. The VE point estimates for all ages were 29% to 61% against any influenza in the primary care setting and 35% to 60% in hospitalised older adults (aged 65 years and over). The VE point estimates against A(H1N1)pdm09 (all ages, both settings) was 48% to 75%, and against A(H3N2) ranged from −58% to 57% (primary care) and −16% to 60% (hospital). Against influenza B, VE for all ages was 62% to 83% (primary care only).

**Conclusions:**

Influenza vaccination is of continued benefit during the ongoing 2019/20 influenza season. Robust end-of-season VE estimates and genetic virus characterisation results may help understand the variability in influenza (sub)type-specific results across studies.

## Introduction

All European Union (EU) countries and the United Kingdom (UK) recommend seasonal influenza vaccine for older adults and those at increased risk of influenza complications and severe disease, as well as for patients with chronic conditions [1]. In addition, universal childhood influenza is available in some countries in the World Health Organization (WHO) European Region, and was introduced incrementally in the UK in 2013/14 [2].

The 2019/20 northern hemisphere influenza season WHO trivalent influenza vaccine strains recommendations were for an A/Brisbane/02/2018 (H1N1)pdm09-like virus, an A/Kansas/14/2017(H3N2)-like virus and a B/Colorado/06/2017-like virus (B/Victoria lineage) [3]. Quadrivalent vaccines were recommended to also include a B/Phuket/3073/2013-like virus (B/Yamagata lineage) [4].

The 2019/20 influenza season started early in most countries of the WHO European Region, with influenza A(H1N1)pdm09 and A(H3N2) virus subtypes, as well as influenza B circulating throughout the region, although predominantly influenza A overall (69%) [5]. Despite this, some countries reported dominance of influenza B, with a few reporting co-dominance [5].

The I-MOVE (Influenza – Monitoring Vaccine Effectiveness in Europe) network has measured influenza vaccine effectiveness (VE) annually since 2008/09, with its partners including Denmark, Spain, the UK and many EU countries measuring VE through the I-MOVE multicentre studies. We summarise interim influenza VE estimates for the 2019/20 season from six studies (four single-country and two multi-country), with out- and in-patient (hospital) settings, in order to provide information for measures of influenza prevention and control for the remaining season. Results presented here also helped to inform the February 2020 WHO Vaccine Strain Selection Committee.

## Methods

### Study setting

**Figure 1:**
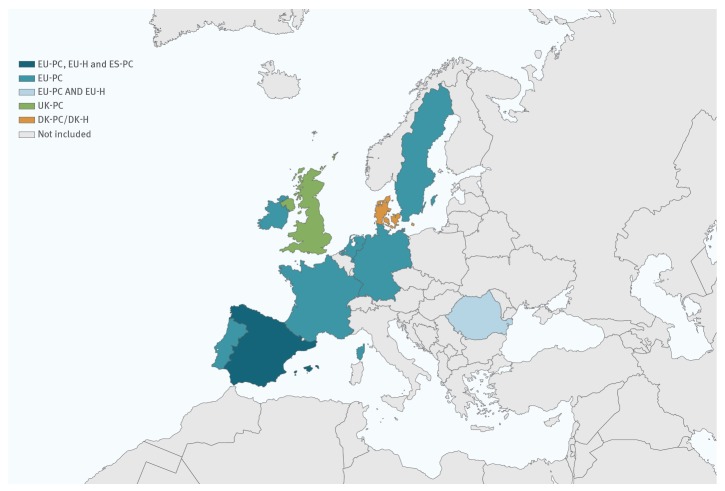
Countries providing interim influenza vaccine effectiveness results, influenza season 2019/20 (n = 10)

The four primary care (PC) studies were conducted in Denmark (DK-PC), Spain (ES-PC), the UK (UK-PC) and through the EU I-MOVE multi-country network (EU-PC; eight of nine participating countries in this network having available data for interim analysis). The two hospital setting (H) studies were in Denmark (DK-H) and through the EU I-MOVE multi-country network (EU-H; two of 11 participating countries in this network having available data for interim analysis) (Figure 1). In total, 10 countries contributed data to the interim influenza vaccine effectiveness results presented in this article.

DK-PC/DK-H: Denmark primary care and hospital studies; ES-PC: Spain primary care study; EU-H: European Union hospital-based multi-country I-MOVE study; EU-PC: European Union primary care-based multi-country I-MOVE study; I-MOVE: Influenza - Monitoring Vaccine Effectiveness in Europe; UK-PC: United Kingdom primary care study.

### Study design

The methods for all six studies have already been described [6-10]. The test-negative design [11] was used in all studies, although some studies varied in their patient selection and/or data collection (Table 1). Briefly, patients presenting to participating primary care settings with symptoms of influenza-like illness (ILI) or acute respiratory infection (ARI) were swabbed. For the hospital setting swabs were taken from those with symptoms of severe ARI (SARI). Three studies used an exhaustive or systematic selection of patients to swab (EU-H, ES-PC and EU-PC), while physicians' discretion was used to select patients for swabbing in the others (DK-H, DK-PC and UK-PC). Samples were tested by reverse transcription (RT)-PCR for generic influenza virus detection, type A subtyping and type B lineage determination. Cases were defined as patients with positive results by influenza virus (sub)type. Controls were defined as those with negative results.

**Table 1:** Summary of methods for the six European interim influenza vaccine effectiveness studies, influenza season 2019/20 (n  = 31,537)

Study characteristics	Study					
	DK-PC	ES-PC	EU-PC	UK-PC	DK-H	EU-H
Study period	1 October 2019–29 January 2020	28 October 2019–26 January 2020	30 September 2019–25 January 2020	1 October 2019–12 January 2020	1 October 2019–29 January 2020	2 December 2019–26 January 2020
Setting	Primary care	Primary care	Primary care	Primary care	Hospital	Hospital
Location	Denmark	Spain: Sentinel networks in 16 of 19 regions	France, Germany, Ireland, the Netherlands, Portugal, Romania, Spain and Sweden	England, Scotland, Northern Ireland and Wales	Denmark	Spain: six hospitals in three regions; Romania: three hospitals
Study design	TND	TND	TND	TND	TND	TND
Data source	Data linkage of Danish Microbiology Database, the Danish Vaccination Register and the Danish National Discharge Register	Sentinel physicians and laboratory^a^	Sentinel physicians and laboratory^a^	Sentinel physicians and laboratory	Data linkage of Danish Microbiology Database, the Danish Vaccination Register and the Danish National Discharge Register	Hospital charts, vaccine registers, interviews with GPs, laboratory
Age groups of study population	≥ 6 months	≥ 6 months	All ages^b^	All ages	≥ 6 months	≥ 65 years
Case definition	Sudden onset of symptoms with fever, myalgia and respiratory symptoms	EU ILI^c^	EU ILI^c^	ILI: Patient presenting in primary care with an acute respiratory illness with physician diagnosed fever, and with onset in the previous 7 days	ARI: Sudden onset of symptoms with fever, myalgia and respiratory symptoms among hospitalised patients	EU SARI^d^
Selection of patients	At physician’s judgement	Systematic	Systematic	At physician’s judgement	At physician’s judgement	Exhaustive
Vaccine types used nationally or in the study^e,f^	In the study: 99.5% QIV, 0.5% cell-propagated QIV	In Spain: 81.8% TIV, 10.3% QIV and 7.8% cell-propagated QIV	In the study among controls: 5% TIV; 2% adjuvanted TIV; 53% QIV; 1% LAIV4; 0% cell-propagated QIV (one person vaccinated among controls), 38% unknown	In the study among controls^g^: 7% LAIV4; 12% cell-propagated QIV, 14% QIV, 22% adjuvanted TIV; 44% unknown	In the study: 99.5% QIV, 0.5% cell-propagated QIV	In the study among controls: 100% TIV; 22% adjuvanted TIV; 1% unknown whether adjuvanted
Variables of adjustment	Age group, sex, presence of chronic conditions, calendar time as month (Oct-Jan)	Age (modelled as RCS or age group depending on analysis), sex, presence of chronic conditions, onset date (RCS), region	Age (modelled as RCS, age group or linear term depending on analysis), sex, presence of any chronic condition associated with influenza vaccination recommendation, onset date (RCS) and study site	Age group, sex, month of onset, surveillance scheme, risk group	Age group, sex, presence of chronic conditions, calendar time as month (Oct-Jan)	Age (modelled as RCS or linear term depending on analysis), sex, presence of any chronic condition associated with influenza vaccination recommendation, onset date (RCS or onset month depending on analysis) and study site

ARI: acute respiratory infection; DK-H: Denmark hospital study; DK-PC: Denmark primary care study; ES-PC: Spain primary care study; EU: European Union; EU-H: EU hospital multicentre I-MOVE study; EU-PC: EU primary care multicentre I-MOVE study; GP: general practitioner; ILI: influenza-like illness; I-MOVE: Influenza – Monitoring Vaccine Effectiveness in Europe; LAIV4: quadrivalent live attenuated influenza vaccine; LRI: lower respiratory infection; QIV: quadrivalent inactivated influenza vaccine; RCS: restricted cubic spline; SARI: severe acute respiratory infection; TIV: trivalent inactivated influenza vaccine; TND: test-negative design; UK-PC: United Kingdom primary care study.

^a^ 235 of 813 sentinel physicians included in ES-PC were also included in EU-PC.

^b^ Patients < 6 months should have been excluded from the study, however the age group is specified as ‘all ages’ as age in months could not be verified from the data.

^c^ EU ILI: Sudden onset of symptoms AND at least one of the following four systemic symptoms: fever or feverishness, malaise, headache, myalgia AND at least one of the following three respiratory symptoms: cough, sore throat, shortness of breath.

^d^ EU SARI: Hospitalised person with one or more of: fever/feverishness, malaise, headache, myalgia, deterioration of general condition (asthenia, weight loss, anorexia, confusion/dizziness) AND one or more respiratory symptom (cough, sore throat or shortness of breath) at admission or within 48 hours.

^e^ Vaccines were prepared from egg-propagated vaccine viruses, non-adjuvanted and administered intramuscularly unless otherwise specified.

^f^ Where indicated, vaccine coverage among controls were used as representative of the source population from which the cases arose.

^g^ UK vaccine strategy: 2–17 years of age: LAIV4 or QIV; 18–64 years of age: QIV or cell-propagated QIV; ≥ 65  years of age: adjuvanted TIV or cell-propagated QIV.

Vaccinated patients were defined as those having had the 2019/20 influenza vaccine at least 14 days before onset of symptoms (15 days for two studies: EU-PC and EU-H). Those vaccinated less than 14 days (less than 15 days for two studies) before symptom onset, or with unknown date of vaccination were excluded.

Most study countries (six from EU-PC and Denmark) selected all or a random sample of influenza virus-positive specimens for haemagglutinin genome segment and/or whole genome sequencing. This was followed by phylogenetic analysis to determine clade distribution for potential impact on VE. In Spain, the ES-PC study (regions not included in EU-PC) sequenced an ad hoc sample of influenza viruses. In UK-PC, sequencing is done by two contributing surveillance schemes. One of these sequences all influenza viruses with sufficient genetic material (Ct value < 31) and all viruses derived from vaccinated cases. The other one sequences a subset only. Sequencing results were provided for both studies in Denmark together (DK-PC and DK-H).

### Ethical statement

The planning, conduct and reporting of the studies was in line with the Declaration of Helsinki [12]. Official ethical approval and patient consent was not required for DK-H and DK-PC according to Danish regulations, nor for EU-PC (the Netherlands and Spain) and UK-PC, as these studies were classified as being part of routine care/surveillance. Other study sites obtained local ethical approval from a national review board, according to local site regulations, as follows: EU-H (Aragon: approved 20 November 2019 by the CEIC Aragon, no registration number given; Granada: approved 11 October 2019 by the CEIM/CEI Provincial de Granada, no registration number given; Navarra: 2018/95; Romania: CE353/30.09.2019); EU-PC (France: 471393; Germany: EA2/126/11; Ireland: ICGP2019.4.04; Portugal: approved 18 January 2012 by the Ethics Committee of Instituto Nacional de Saúde Doutor Ricardo Jorge, no registration number given; Romania: CE354/30.09.2019; Sweden: 2006/1040–31/2).

### Statistical analysis

Each study computed VE by subtracting the ratio of the odds of vaccination in cases and controls from one, as a percentage (VE = (1 – odds ratio (OR) x 100%). All studies applied logistic regression to adjust their VE for measured confounding variables (Table 1). Study-specific VE was estimated overall and where possible, by age group and target population (as defined locally in the various studies and study sites) against influenza A overall, A(H1N1)pdm09, A(H3N2), influenza B and B/Victoria. For analyses with a small sample size, defined as those having fewer than 10 cases or controls per parameter, a sensitivity analysis was performed using Firth’s method of penalised logistic regression (PLR) to assess small sample bias [13,14]. We considered a difference of > 10% between the PLR and original estimate to be an indication of small sample bias, and any such estimates were not included.

## Results

From 30 September 2019 to 29 January 2020, in the primary care setting, there were 12,842 patients included in the DK-PC study (1,723 cases), 1,798 in ES-PC (955 cases), 2,987 in EU-PC (1,052 cases) and 2,548 in UK-PC (782 cases). In the hospital setting, there were 10,761 in DK-H (659 cases) and 601 in EU-H (129 cases).

**Figure 2:**
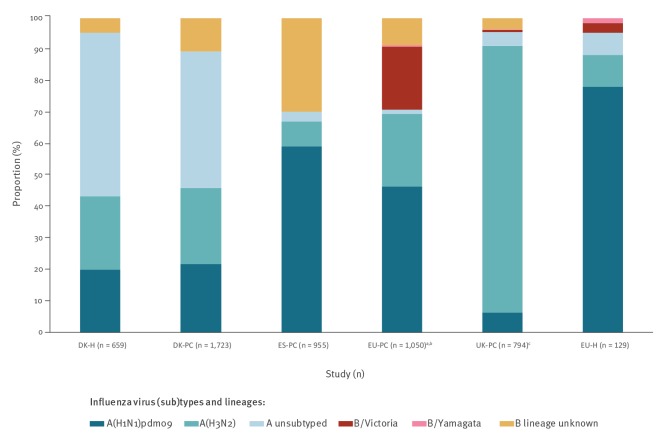
Proportion of influenza virus (sub)types among cases, six European studies, interim influenza season 2019/20 (n = 5,310)

DK-H: Denmark hospital study; DK-PC: Denmark primary care study; ES-PC: Spanish primary care study; UK-PC: United Kingdom primary care study; EU-H: European Union hospital multicentre I-MOVE study; EU-PC: European Union primary care multicentre I-MOVE study; I-MOVE: Influenza - Monitoring Vaccine Effectiveness in Europe.

^a^ 246 of influenza A(H1N1)pdm09, eight A(H3N2) and 79 B cases are also in ES-PC.

^b^ Includes three co-infections. These, and two patients from one site, were not included in the A(H3N2) analysis, but in the ‘any influenza A’ analysis.

^c^ Includes 12 co-infections.

Overall, 84% (4,466/5,310) of confirmed infections were influenza A virus-positive and 16% (844/5,310) were influenza B virus-positive, noting that the previously mentioned total refers to patients rather than infections. The proportion of subtyped influenza A viruses was 93% to 98% in EU-H, ES-PC, EU-PC and UK-PC, and 45% to 51% in DK-H/DK-PC. Most subtyped influenza A viruses were influenza A(H1N1)pdm09 (67–89%) in ES-PC, EU-PC and EU-H; this subtype comprised 46% to 47% in DK-PC/DK-H and 7% in UK-PC (Figure 2). The proportion of influenza B viruses ranged from 4% in UK-PC to 30% in ES-PC (Figure 2). The proportion of B viruses ascribed to a lineage was 27% overall (100% for EU-H, 70% for EU-PC, 23% for UK; lineage information not available in DK-PC, DK-H and ES-PC studies). Most of those that were ascribed to a lineage were B/Victoria: 98% in EU-PC, 75% in UK and 67% in EU-H.

### All influenza (A and B)

#### Primary care settings

For all ages, VE against laboratory-confirmed influenza (both A and B combined) ranged from 29% (95% confidence interval (CI): 4 to 48) in UK-PC to 61% (95% CI: 37 to 76) in ES-PC. The VE against all influenza among children aged 0 to 17 years was 64% (95% CI: 16 to 85) in EU-PC and 95% (95% CI: 67 to 99) in DK-PC. For those aged 0 to 14 years in ES-PC, VE was 67% (95% CI: 18 to 87), while for those aged 2 to 17 years in UK-PC, VE was 37% (95% CI: −21 to 67). For patients aged 18 to 64 years, VE ranged from 36% (95% CI: 1 to 58) in UK-PC to 58% (95% CI: 43 to 69) in DK-PC. For those aged 15 to 64 years in ES-PC, VE was 55% (95% CI: 10 to 77) (Table 2). In ES-PC and EU-PC target groups for influenza vaccination, VE was 60% (95% CI: 22 to 79) and 53% (95% CI: 26 to 70), respectively.

**Table 2:** Interim vaccine effectiveness (VE) against all laboratory-confirmed influenza, influenza A, A(H1N1)pdm09, A(H3N2) and B, by age group, target group for vaccination and by study, six European studies, influenza season 2019/20

Influenza (sub)type and study	Setting	Study population^a^	Cases			Controls			VE^b^	95% CI
			All	Vacc	%	All	Vacc	%		
**All influenza (A and B)**										
DK-PC^c^	PC	All ages	1,715	119	7	11,127	1,349	12	56	46 to 65
		0–17 years	669	1	< 1	3,793	669	18	95	67 to 99
		18–64 years	862	48	6	5,436	480	9	58	43 to 69
		≥ 65 years	184	70	38	1,898	808	43	40	18 to 57
ES-PC	PC	All ages	955	46	5	843	79	9	61	37 to 76
		0–14 years	416	14	3	298	15	5	67	18 to 87
		15–64 years	513	19	4	473	27	6	55	10 to 77
		Target group^d^	105	27	26	181	57	31	60	22 to 79
EU-PC	PC	All ages	1,052	76	7	1,935	211	11	53	34 to 67
		0–17 years	419	12	3	810	28	4	64	16 to 85
		18–64 years	566	33	6	920	81	9	51	21 to 70
		Target group^d^	197	52	26	509	158	31	53	26 to 70
UK-PC	PC	All ages	782	114	15	1,766	308	17	29	4 to 48
		2–17 years	303	24	8	358	28	8	37	−21 to 67
		18–64 years	379	44	12	937	119	13	36	1 to 58
		≥ 65 years	72	46	64	281	161	57	26	−44 to 62
DK-H^c^	H	All ages	658	168	26	10,103	2,745	27	40	27 to 51
		18–64 years	237	30	13	3,397	533	16	48	22 to 65
		≥ 65 years	315	134	43	5,008	2,166	43	35	17 to 49
EU-H^c^	H	≥ 65 years	128	54	42	473	312	66	60	39 to 74
**Influenza A**										
DK-PC	PC	All ages	1,540	115	8	11,127	1,349	12	54	43 to 63
		0–17 years	579	1	< 1	3,793	61	2	95	63 to 99
		18–64 years	786	47	6	5,436	480	9	54	38 to 67
		≥ 65 years	175	67	38	1,898	808	43	41	18 to 57
ES-PC	PC	All ages	670	40	6	843	79	9	60	34 to 76
		0–14 years	244	12	5	298	15	5	48	−39 to 81
		15–64 years	400	15	4	473	27	6	62	20 to 82
		Target group^d^	90	23	26	181	57	31	62	24 to 82
EU-PC	PC	All ages	746	67	9	1,906	210	11	53	32 to 67
		0–17 years	261	10	4	800	28	4	53	−19 to 81
		18–64 years	426	27	6	903	81	9	60	32 to 76
		Target group^d^	161	46	29	503	157	31	49	16 to 69
UK-PC	PC	All ages	756	110	15	1,766	308	17	30	4 to 49
		2–17 years	294	23	8	358	28	8	39	−19 to 69
		18–64 years	364	42	12	937	119	13	38	3 to 60
		≥ 65 years	70	45	64	281	161	57	24	−49 to 61
DK-H	Hospital	All ages	629	162	26	10,103	2,745	27	41	27 to 52
		18–64 years	226	29	13	3,397	533	16	47	21 to 65
		≥ 65 years	306	129	42	5,008	2,166	43	37	19 to 50
EU-H^c^	Hospital	≥ 65 years	122	50	41	473	312	66	62	41 to 76
**Influenza A(H1N1)pdm09**										
DK-PC	PC	All ages	373	14	4	11,127	1,349	12	75	57 to 86
		18–64 years	229	10	4	5,436	480	9	67	38 to 83
		≥ 65 years	28	4	NC	1,898	808	43	79	37 to 93
ES-PC	PC	All ages	566	30	5	843	79	9	69	46 to 82
		0–14 years	198	10	5	298	15	5	51	−45 to 83
		15–64 years	349	12	3	473	27	6	68	28 to 86
		Target group^d^	76	17	22	181	57	31	73	40 to 88
EU-PC	PC	All ages	487	33	7	1,906	210	11	48	18 to 68
		0–17 years	172	9	5	786	27	3	46	−51 to 80
		18–64 years	292	14	5	903	81	9	49	1 to 74
DK-H	Hospital	All ages	132	22	17	10,103	2,745	27	54	24 to 72
		18–64 years	68	8	12	3,397	533	16	55	3 to 79
		≥ 65 years	44	12	NC	5,008	2,166	43	51	4 to 75
EU-H^c^	Hospital	≥ 65 years	98	42	43	445	303	68	63	40 to 77
**Influenza A(H3N2)**										
DK-PC	PC	All ages	418	45	11	11,127	1,349	12	27	−4 to 49
		18–64 years	190	17	9	5,436	480	9	29	−19 to 57
		≥ 65 years	55	27	NC	1,898	808	43	12	−53 to 49
ES-PC	PC	All ages	75	10	13	799	79	10	−58	−338 to 43
EU-PC	PC	All ages	244	33	14	1,772	180	10	57	27 to 75
		18–64 years	125	12	10	834	75	9	71	37 to 87
		Target group^d^	70	28	40	431	126	29	38	−21 to 69
UK-PC	PC	All ages	675	103	15	1,766	308	17	25	−3 to 46
		2–17 years	273	22	8	358	28	8	39	−21 to 69
		18–64 years	308	38	12	937	119	13	31	−8 to 56
		≥ 65 years	66	43	65	281	161	57	21	−56 to 60
DK-H	Hospital	All ages	154	59	38	10,103	2,745	27	−13	−58 to 19
		≥ 65 years	89	53	60	5,008	2,166	43	−16	−80 to 25
EU-H^c^	Hospital	≥ 65 years	12	4	NC	313	199	64	60	−69 to 90
**Influenza B**										
DK-PC	PC	All ages	183	4	2	11,127	1,349	12	83	51 to 94
ES-PC	PC	All ages	285	6	2	843	79	9	66	7 to 87
EU-PC	PC	All ages	305	9	3	1,373	169	12	62	17 to 83
		18–64 years	138	6	4	658	64	10	12	−135 to 67
**Influenza B Victoria**										
EU-PC^c^	PC	All ages	209	5	2	1,190	141	12	60	−12 to 86

CI: confidence interval; DK-PC: Denmark primary care study; DK-H: Denmark hospital study; ES-PC: Spain primary care study; EU-H: European Union hospital multicentre I-MOVE study; EU-PC: European Union primary care multicentre I-MOVE study; I-MOVE: Influenza - Monitoring Vaccine Effectiveness in Europe; LAIV4: quadrivalent live attenuated influenza vaccine; NC: not calculated (percentages not shown where denominators < 60); PC: primary care; UK-PC: United Kingdom primary care study; Vacc: vaccinated; VE: vaccine effectiveness.

Countries included in EU-H analysis for any influenza, influenza A and influenza A(H3N2): Romania and Spain. For analysis against influenza A(H1N1)pdm09: Spain only.

Countries included in EU-PC analysis for all influenza, influenza A and A(H1N1)pdm09: France, Germany, Ireland, the Netherlands, Portugal, Romania, Spain and Sweden. For analysis against influenza A(H3N2): France, Germany, Ireland, the Netherlands, Romania, Spain and Sweden are included. For analysis against influenza B: France, Germany, Ireland, Portugal, Romania, Spain and Sweden. For analysis against influenza B/Victoria: France, Germany, Ireland, Portugal, Romania and Sweden.

^a^ Age-specific or target group-specific VE was not included for overall or (sub)type-specific VE in some study sites, where sample size did not allow estimation of VE.

^b^ For details of adjustment variables, see Table 1.

^c^ Some records with missing values were dropped from this analysis.

^d^ Groups targeted by seasonal influenza vaccination as defined locally in the studies and study sites.

#### Hospital settings

For all ages, VE against all laboratory-confirmed hospitalised influenza was 40% in DK-H (95% CI: 27 to 51). In older adults (aged at least 65 years), VE was 35% (95% CI: 17 to 49) in DK-H and 60% (95% CI: 39 to 74) in EU-H.

### Influenza A overall

#### Primary care settings

The VE against laboratory-confirmed influenza A for all ages ranged from 30% (95% CI: 4 to 49) in UK-PC to 60% (95% CI: 34 to 76) in ES-PC. The VE against influenza A among adults under 65 years ranged from 38% (95% CI: 3 to 60) in UK-PC (18–64-year-olds) to 62% (95% CI: 20 to 82) in the ES-PC study (15–64-year-olds). In children less than 18 years of age, the VE ranged from 39% (95% CI: -19 to 69) in UK-PC (2–17-year-olds) to 95% (95% CI: 63 to 99) in DK-PC (0–17-year-olds) (Table 2).The VE in EU-PC and ES-PC target groups for influenza vaccination was 49% (95% CI: 16 to 69) and 62% (95% CI: 24 to 82), respectively.

#### Hospital settings

For all ages, VE against laboratory-confirmed hospitalised influenza A was 41% (95% CI: 27 to 52) in DK-H. For older adults, VE was 37% (95% CI: 19 to 50) in DK-H and 62% (95% CI: 41 to 76) in EU-H.

### Influenza A(H1N1)pdm09

#### Primary care settings

For all ages, VE against laboratory-confirmed influenza A(H1N1)pdm09 ranged between 48% (95% CI: 18 to 68) in EU-PC and 75% (95% CI: 57 to 86) in DK-PC. The number of vaccinated cases in the UK-PC study was too small to provide a VE estimate.

The VE among children less than 18 years of age was 46% (95% CI: −51 to 80) in EU-PC (0–17 years) and 51% (95% CI −45 to 83) in ES-PC (0–14 years). Among adults less than 65 years, VE was between 49% (95% CI: 1 to 74) in EU-PC (18–64 years) and 68% (95% CI: 28 to 86) in ES-PC (15–64 years). VE for adults aged 65 years and older was 79% (95% CI: 37 to 93) in the DK-PC study. Target groups in the ES-PC had VE of 73% (95% CI: 40 to 88) against influenza A(H1N1)pdm09.

#### Hospital settings

For hospitalised patients aged 65 years and older, VE was 51% (95% CI: 4 to 75) in DK-H and 63% (95% CI: 40 to 77) in the EU-H study (Table 2). For hospitalised patients in DK-H aged 18 to 64 years, VE was 55% (95% CI: 3 to 79).

#### Virological results

Among the 212 A(H1N1)pdm09 viruses sequenced, 90% (n = 190) belonged to genetic clade 6B.1A5A (Table 3) and none belonged to the same clade as the vaccine component (6B.1A1). Twenty viruses (9%) belonged to the 6B.1A5B clade and one virus (1%) each to the 6B.1A6 and 6B.1A7 clades.

**Table 3:** Influenza viruses characterised by clade, amino acid substitutions and study site, five European studies, interim influenza season 2019/20 (n = 605)

Influenza virus	Clade	DK-H/DK-PC^a^		ES-PC^b^		EU-PC^c,d^		UK-PC^d^	
		n	%	n	%	n	%	n	%
**Total influenza A(H1N1)pdm09**		**n = 505**		**n = 566**		**n = 496**		**n = 50**	
**Sequenced**		**72**	15	**86**	15	**46**	9	**8**	16
A/Norway/3433/2018-like	6B.1A5A	56	78	84	98	43	NC	7	NC
A/Switzerland/3330/2017-like	6B.1A5B	15	21	2	2	3	NC	0	NC
A/Slovenia/1489/2019-like	6B.1A7	1	1	0	0	0	NC	0	NC
A/Brisbane/02/2018-like	6B.1A1	0	0	0	0	0	NC	0	NC
A/Ireland/84630/2018-like	6B.1A6	0	0	0	0	0	NC	1	NC
**Total influenza A(H3N2)**		**n = 572**		**n = 75**		**n = 250**		**n = 675**	
**Sequenced**		68	12	23	31	75	30	168	25
A/South Australia/34/2019-like	3C.2a1b + T131K	27	40	6	NC	21	28	13	8
A/La Rioja/2202/2018-like	3C.2a1b + T135K-A	1	1	9	NC	10	13	1	1
A/Hong Kong/2675/2019-like	3C.2a1b + T135K-B	9	13	1	NC	1	1	4	2
A/Kansas/14/2017-like	3C.3a	31	46	7	NC	43	57	150	89
**Total influenza B**		**n = 213**		**n = 285**		**n = 317**		**n = 8**	
**Sequenced^e^**		8	4	0	0	46	15	5	63
**Total B/Victoria among sequenced**		8	NC	0	NC	46	NC	5	NC
B/Colorado/06/2017-like	1A (del162–163)	0	NC	0	NC	1	NC	0	NC
B/Hong Kong/269/201-like	1A (del162–164)A	0	NC	0	NC	0	NC	0	NC
B/Washington/02/2019-like	1A (del162–164)B	8	NC	0	NC	45	NC	5	NC

DK-PC: Denmark primary care study; DK-H: Denmark hospital study; ES-PC: Spain primary care study; EU-PC: European Union primary care multicentre I-MOVE study; I-MOVE: Influenza – Monitoring Vaccine Effectiveness in Europe; NC: not calculated (percentages not shown where denominators < 60); UK-PC: United Kingdom primary care study.

^a^ DK-H and DK-PC samples are combined; sequence information is based on influenza-positive samples received for surveillance at the National Influenza Center Denmark from week 40/2019 and 4/2020.

^b^ Specimens sequenced from Spain originate from the entire National Influenza Surveillance System in weeks 40/2019 to 4/2020.

^c^ Two specimens from ES-PC also included in EU-PC data.

^d^ At time of publishing not all specimens from the study period were processed.

^e^ No B/Yamagata samples had been sequenced at the time of publishing.

### Influenza A(H3N2)

#### Primary care settings

For patients of all ages, VE against influenza A(H3N2) ranged from −58% (95% CI: −338 to 43) in ES-PC to 57% (95% CI: 27 to 75) in EU-PC. For children aged 2 to 17 years in the UK-PC, VE was 39% (95% CI: −21 to 69). For those aged 18 to 64 years, VE was between 29% (95% CI: −19 to 57) in DK-PC and 71% (95% CI: 37 to 87) in EU-PC. VE ranged from 12% (95% CI: −53 to 49) in DK-PC to 21% (95% CI: −56 to 60) in UK-PC in those aged 65 years and over. For target groups in the EU-PC, VE against influenza A(H3N2) was 38% (95% CI: −21 to 69) (Table 2).

#### Hospital settings

VE among hospitalised patients aged 65 years and older against influenza A(H3N2) was between −16% (95% CI: −80 to 25) in DK-H and 60% (95% CI: −69 to 90) in EU-H (Table 2).

#### Virological results

Of the 334 influenza A(H3N2) viruses sequenced, 69% (n = 231) belonged to the same genetic clade as the vaccine strain (3C.3a), 20% (n = 67) belonged to 3C.2a1b + T131K, 6% to 3C.2a1b + T135K-A (n = 21) and 5% to 3C.2a1b + T135K-B (n = 15). The distribution of (sub)clades varied by country, e.g. from 46% (n = 31) in DK-H/DK-PC to 89% (n = 150) in UK-PC for clade 3C.3a, and with only 23 of 75 A(H3N2) viruses sequenced overall in ES-PC.

### Influenza B

#### Primary care settings

Sample sizes for influenza B were large enough to estimate VE in three studies in the primary care setting only (DK-PC, ES-PC and EU-PC). The VE against laboratory-confirmed influenza B for all age groups ranged from 62% (95% CI: 17 to 83) in EU-PC to 83% (95% CI: 51 to 94) in DK-PC. For those aged 18–64 years, VE was 12% in EU-PC (95% CI: −135 to 67) (Table 2). VE against B/Victoria was 60% (95% CI: −12 to 86) (only estimated for EU-PC). The number of cases of B/Yamagata was too low for VE to be estimated.

#### Virological results

Of the 59 influenza B/Victoria viruses sequenced, 58 belonged to subgroup 1A(del162–164)B and one belonged to the vaccine subclade 1A(del162–163).

### Sensitivity analyses

Results with small sample sizes were subject to sensitivity analyses, all of which gave similar results (absolute difference range: 1–6%).

## Discussion

Our results for the 2019/20 influenza season in six well-known influenza studies across Europe indicate that interim VE against any laboratory-confirmed influenza among all ages in primary care and hospital settings ranged between 29% and 61%, while VE was from 53% to 60% in vaccination target groups. The proportions of influenza (sub)types contributing to these overall results varied considerably by study.

Against influenza A (all subtypes) among all ages, point estimates for VE ranged between 30% and 60% in both types of settings, and they were slightly higher (49–62%) in the target groups for vaccination. Against influenza A(H1N1)pdm09, VE point estimates among all ages ranged from 48% to 75%, being slightly higher among older adults (aged 65 years and older) in DK-PC at 79% and slightly lower in children aged 0 to 17 years in EU-PC (46%). There were varied results for VE against influenza A(H3N2). For patients of all ages combined, two studies (one primary care, one hospital) had VE point estimates < 0%, two (primary care) had VE < 30%, and two (one primary care, one hospital) had VE > 50%. The VE point estimate against A(H3N2) was highest among 18 to 64-year-olds in EU-PC (71%). Against laboratory-confirmed influenza B, VE among all ages ranged from 62% to 83% in primary care settings.

The VE point estimates against all influenza from three of four primary care studies (DK-PC, ES-PC and EU-PC) over all ages, at 53% to 61%, are similar to VE point estimates from Canada (58%) [15], and a little higher than those reported from the United States (US) (45%) [16], noting that proportions of influenza (sub)types and proportions of study participants contributing to age groups may be slightly different across all studies. For older adults (aged 65 years and over), the six studies presented here had VE point estimates of 43% to 66% across all settings. This is similar to estimates from Finland, Sweden and Canada for this age group, at 41% [17], 44% [18] and 60% [15], respectively. It is also similar for those aged 50 years and over in the US, at 43% [16], noting that underlying proportions of influenza (sub)types are likely to be different across all studies.

The 2019/20 interim VE against influenza A(H1N1)pdm09 was higher compared with the 2018/19 interim season estimates in most studies among all ages, except for the EU-PC study [19]. The VE against A(H1N1)pdm09 was also higher among those aged 65 years and older in the hospital-based studies in the 2019/20 season compared with the previous season [19]. The main circulating genetic clade in the 2019/20 studies is 6B.1A5A and to a lesser extent, 6B.1A5B, which although different from the vaccine strain, show good reactivity with ferret antiserum raised against the vaccine virus [20]. The 2019/20 interim influenza A(H1N1)pdm09 VE in Canada [15] and the US [16] was 44% and 37%, respectively. These overall results are lower than the DK-PC and ES-PC results, but are more comparable with the EU-PC results (48%). In the US, there was some indication of lower VE among younger adults (18–49 years) with VE being 5% [16]. None of the studies reported in this article indicate a much lower A(H1N1)pdm09 VE among younger adults. End-of-season overall results as well as clade/genetic variant-specific results and birth cohort-specific VE will help understand differences between studies at international level.

Unlike observed estimates in the 2018/19 season, in which three of five studies reporting A(H3N2) cases had VE below zero and all were below 50% [19], two of six 2019/20 interim study results were below zero, two were between 25% and 50%, and two were between 50% and 60%. The EU-PC and the ES-PC studies showed very different point estimates of A(H3N2) VE among all ages; however, there were only eight cases in common across these studies. There is considerable variation in the VE estimates against A(H3N2) presented here, which may go hand-in-hand with the considerable genetic diversity observed. While A(H3N2) viruses remain difficult to characterise antigenically [20], reports suggest that the 3C.3a circulating viruses were antigenically similar to the vaccine virus. While a higher VE against A(H3N2) among studies with high proportions of 3C.3a characterised viruses might be anticipated, this was not observed everywhere. The other viruses circulating belonged to the 3C.2a1b clade, most with additional substitutions. These viruses are more genetically distinct from the vaccine virus and therefore antigenically less similar. However, 2019/20 interim VE from Canada against A(H3N2) was 62%, and 94% of the 80 viruses sequenced in Canada belonged to the 3C.2a1b clade. While clade-specific VE estimates at the end of the 2019/20 season could elucidate whether different circulating clades of A(H3N2) viruses across the region explain the differences in VE against A(H3N2) observed here in these studies, some of the observed differences in interim VE may indicate that further explanations, such as immunological cohort effects [21], need to be investigated.

Recent vaccine seed A(H3N2) viruses have developed adaptions during propagation in eggs, potentially negatively affecting VE [22]. Cell-propagated vaccines were used in some studies, but there was insufficient data to estimate vaccine type-specific VE.

VE against influenza B was > 60% among all ages in primary care, with a lower VE among those aged 18 to 64 years in EU-PC. However, sample size was lower in this age group and the low VE may possibly be a result of random variation. The high overall VE was comparable with 2019/20 interim VE against influenza B in Canada (69%) [15]. In the US, overall VE against B/Victoria was lower, at 50% [16].

In studies where lineage was available, the vast majority of circulating influenza B belonged to the B/Victoria lineage, which was the lineage included in the trivalent vaccine but of another subgroup. The quadrivalent vaccine was used widely in Europe, however because of low circulation of B/Yamagata viruses, the VE of quadrivalent and trivalent vaccines could not be compared in the context of VE against trivalent lineage-mismatched influenza B. Among the sequenced B/Victoria viruses, 98% belonged to the subgroup 1A(del162–164)B, differing from the vaccine virus by a further amino acid deletion, and being antigenically different [3,23,24]. Nevertheless, human serology studies show some evidence of cross-reactivity between the vaccine and the circulating B/Victoria viruses [20,23,24].

The early start of the season in most European countries included in these six studies [5] resulted in higher incidence and greater precision for interim VE estimates than in 2018/19, although some studies did have lower sample size for some subgroups in this interim analysis. Each study used study-specific criteria to define if a sample size was too small to attempt to estimate VE. Where VE results were presented, and sample size was small, sensitivity analyses were used to address potential small sample bias where appropriate. End-of-season estimates will have higher sample size and provide more robust estimates. Residual confounding and bias are known limitations potentially present in all observational studies.

Vaccination remains the key successful method of influenza prevention, with one to two-thirds of all vaccinated individuals receiving protection during the 2019/20 influenza season. Promotion of influenza vaccination should be maintainted in line with national guidelines and recommendations in all European countries with ongoing influenza virus circulation. Given the variation in VE estimates against A(H3N2), it remains important that when national guidelines indicate neuraminidase inhibitors to be used, they are used regardless of vaccination status as prophylaxis and therapy where there is influenza A(H3N2) virus circulation to help prevent severe outcomes [1].

Bi-annual reports on influenza VE in prior and existing seasons are provided by the Global Influenza VE (GIVE) Collaboration. The February 2020 GIVE report included interim VE results presented here to help inform the WHO Vaccine Strain Selection Committee meeting of 24 to 27 February 2020 in Geneva. For the 2020/21 northern hemisphere season, WHO provided specific recommendations for egg-based, cell-based and recombinant-based vaccines [25]. Compared with the northern hemisphere 2019/20 trivalent vaccine recommendations, all components for the 2020/21 trivalent vaccine have been changed. For influenza A(H1N1)pdm09, WHO recommended A/Guangdong-Maonan/SWL1536/2019(H1N1)pdm09-like virus for egg-based vaccines and A/Hawaii/70/2019 (H1N1)pdm09-like virus for cell-based or recombinant-based vaccines, which are 6B.1A5A viruses, harbouring additional D187A and Q189E substitutions. For influenza A(H3N2), WHO recommended A/HongKong/2671/2019 (H3N2)-like virus for egg-based vaccines and A/HongKong/45/2019 (H3N2)-like virus for cell-based or recombinant-based vaccines, both 3C.2a1b + T135K-B viruses, harbouring S137F, A138S and F193SHA substitutions. For the 2020 southern hemisphere influenza vaccine, WHO recommended for both 2020/21 northern hemisphere trivalent and quadrivalent vaccines a B/Washington/02/2019-like (B/Victoria lineage) virus (a three amino acid deletion virus), and additionally, a B/Phuket/3073/2013-like (B/Yamagata lineage) virus for the quadrivalent vaccine [25].

The VE and antigenic studies at the end of the 2019/20 season will help to explain the differences in age-, subtype- and study-specific VE estimates presented here. In order to be prepared for the next season in the northern hemisphere, we should continue to monitor the genetic diversity of the 2020 southern hemisphere influenza viruses and their influenza VE.

## European IVE group

Denmark

DK-H and DK-PC studies:

Hanne-Dorthe Emborg, Department of Infectious Disease Epidemiology and Prevention, Statens Serum Institut, Copenhagen

Ramona Trebbien, Department of Virus and Microbiological Special diagnostics, National Influenza Center, Statens Serum Institut, Copenhagen

Daniel Thomas Lopez, Department of Virus and Microbiological Special diagnostics, National Influenza Center, Statens Serum Institut, Copenhagen; European Public Health Microbiology Training Programme (EUPHEM), European Centre for Disease Prevention and Control (ECDC), Stockholm, Sweden

Tyra Grove Krause, Department of Infectious Disease Epidemiology and Prevention, Statens Serum Institut, Copenhagen

France

EU-PC study:

Alessandra Falchi, EA 7310, Laboratoire de Virologie, Université de Corse-Inserm, Corte

Shirley Masse, EA 7310, Laboratoire de Virologie, Université de Corse-Inserm, Corte

Natacha Villechenaud, EA 7310, Laboratoire de Virologie, Université de Corse-Inserm, Corte

Ana-Maria Vilcu, INSERM, Sorbonne Université, Institut Pierre Louis d’Epidémiologie et de Santé Publique (IPLESP UMRS 1136), Paris

Cécile Souty, Sorbonne Université, INSERM, Institut Pierre Louis d’Epidémiologie et de Santé Publique (IPLESP UMRS 1136), Paris

Thierry Blanchon, Sorbonne Université, INSERM, Institut Pierre Louis d’Epidémiologie et de Santé Publique (IPLESP UMRS 1136), Paris

Titouan Launay, Sorbonne Université, INSERM, Institut Pierre Louis d’Epidémiologie et de Santé Publique (IPLESP UMRS 1136), Paris

Sylvie van der Werf, Unité de Génétique Moléculaire des Virus à ARN, UMR 3569 CNRS, Université de Paris, Institut Pasteur, Paris; CNR des virus des infections respiratoires, Institut Pasteur, Paris

Vincent Enouf, Unité de Génétique Moléculaire des Virus à ARN, UMR 3569 CNRS, Université de Paris, Institut Pasteur, Paris; CNR des virus des infections respiratoires, Institut Pasteur, Paris

Sylvie Behillil, Unité de Génétique Moléculaire des Virus à ARN, UMR 3569 CNRS, Université de Paris, Institut Pasteur, Paris; CNR des virus des infections respiratoires, Institut Pasteur, Paris.

Bruno Lina, Laboratoire de Virologie, CNR des virus des infections respiratoires, Institut des Agents Infectieux, Groupement Hospitalier Nord des HCL, Lyon, France; Laboratoire Virpath, CIRI Inserm U1111, CNRS 5308, ENS, UCBL, Faculté de Médecine Lyon Est, Université de Lyon, Lyon

Martine Valette, Laboratoire de Virologie, CNR des virus des infections respiratoires, Institut des Agents Infectieux, Groupement Hospitalier Nord des HCL, Lyon

Epiconcept

EU-PC and EU-H studies:

Marta Valenciano, Alain Moren, Epiconcept, Paris, France

EU-PC study:

Esther Kissling, Epiconcept, Paris, France

EU-H study:

Angela Rose, Epiconcept, Paris, France

European Centre for Disease Prevention and Control

EU-PC study:

Pasi Penttinen, ECDC, Stockholm, Sweden

Germany

EU-PC study:

Silke Buda, Department for Infectious Disease Epidemiology, Respiratory Infections Unit, Robert Koch Institute, Berlin

Ute Preuss, Department for Infectious Disease Epidemiology, Respiratory Infections Unit, Robert Koch Institute, Berlin

Kristin Tolksdorf, Department for Infectious Disease Epidemiology, Respiratory Infections Unit, Robert Koch Institute, Berlin

Barbara Biere, National Reference Center for Influenza, Robert Koch Institute, Berlin

Ralf Duerrwald, National Reference Center for Influenza, Robert Koch Institute, Berlin

Maria Smallfield, National Reference Center for Influenza, Robert Koch Institute, Berlin

Marianne Wedde, National Reference Center for Influenza, Robert Koch Institute, Berlin

Ireland

EU-PC study:

Lisa Domegan, HSE-Health Protection Surveillance Centre, Dublin; European Programme for Intervention Epidemiology Training (EPIET), European Centre for Disease Prevention and Control (ECDC), Stockholm, Sweden

Rachel Fiegenbaum, HSE-Health Protection Surveillance Centre, Dublin

Joan O’Donnell, HSE-Health Protection Surveillance Centre, Dublin

Michael Joyce, Irish College of General Practitioners, Dublin

Olga Levis, Irish College of General Practitioners, Dublin

Claire Collins, Irish College of General Practitioners, Dublin

Charlene Bennett, National Virus Reference Laboratory, University College Dublin, Dublin

Joanne Moran, National Virus Reference Laboratory, University College Dublin, Dublin

Grainne Tuite, National Virus Reference Laboratory, University College Dublin, Dublin

Jeff Connell, National Virus Reference Laboratory, University College Dublin, Dublin

Cillian de Gascun, National Virus Reference Laboratory, University College Dublin, Dublin

The Netherlands

EU-PC study:

Adam Meijer, National Institute for Public Health and the Environment (RIVM), Bilthoven

Marit de Lange, National Institute for Public Health and the Environment (RIVM), Bilthoven

Sharon van den Brink, National Institute for Public Health and the Environment (RIVM), Bilthoven

Frederika Dijkstra, National Institute for Public Health and the Environment (RIVM), Bilthoven

Mariette Hooiveld, Nivel (the Netherlands Institute for Health Services Research), Utrecht

Portugal

EU-PC study:

Ana Paula Rodrigues, Departamento de Epidemiologia, Instituto Nacional de Saúde Dr Ricardo Jorge, Lisbon

Ausenda Machado, Departamento de Epidemiologia, Instituto Nacional de Saúde Dr Ricardo Jorge, Lisbon

Baltazar Nunes, Departamento de Epidemiologia, Instituto Nacional de Saúde Dr Ricardo Jorge, Lisbon

Irina Kislaya, Departamento de Epidemiologia, Instituto Nacional de Saúde Dr Ricardo Jorge, Lisbon

Verónica Gomez, Departamento de Epidemiologia, Instituto Nacional de Saúde Dr Ricardo Jorge, Lisbon

Inês Costa, Departamento de Doenças Infeciosas, Instituto Nacional de Saúde Dr Ricardo Jorge, Lisbon

Patrícia Conde, Departamento de Doenças Infeciosas, Instituto Nacional de Saúde Dr Ricardo Jorge, Lisbon

Paula Cristóvão, Departamento de Doenças Infeciosas, Instituto Nacional de Saúde Dr Ricardo Jorge, Lisbon

Pedro Pechirra, Departamento de Doenças Infeciosas, Instituto Nacional de Saúde Dr Ricardo Jorge, Lisbon

Raquel Guiomar, Departamento de Doenças Infeciosas, Instituto Nacional de Saúde Dr Ricardo Jorge, Lisbon

Romania

EU-PC and EU-H studies:

Mihaela Lazar, “Cantacuzino” National Medico-Military Institute for Research and Development, Bucharest

Carmen Maria Cherciu, “Cantacuzino” National Medico-Military Institute for Research and Development, Bucharest

Maria Elena Mihai, “Cantacuzino” National Medico-Military Institute for Research and Development, Bucharest

EU-H study:

Elena Duca, Clinical Hospital of Infectious Diseases “Sf Parascheva”, Iasi

Mihaela Catalina Luca, Clinical Hospital of Infectious Diseases “Sf Parascheva”, Iasi

Maria Gradinaru, Clinical Hospital of Infectious Diseases “Sf Parascheva”, Iasi

Izabela Loghin, Clinical Hospital of Infectious Diseases “Sf Parascheva”, Iasi

Carmen Manciuc, Clinical Hospital of Infectious Diseases “Sf Parascheva”, Iasi

Grațiela Tardei, Clinical Hospital of Infectious Diseases “Dr Victor Babes”, Bucharest

Maria Nica, Clinical Hospital of Infectious Diseases “Dr Victor Babes”, Bucharest

Simin-Aysel Florescu, Clinical Hospital of Infectious Diseases “Dr Victor Babes”, Bucharest

Emanoil Ceausu, Clinical Hospital of Infectious Diseases “Dr Victor Babes”, Bucharest

Spain

EU-PC (the cycEVA work group), ES-PC (the Spanish Influenza Sentinel Surveillance System) and EU-H studies:

Amparo Larrauri, National Centre of Epidemiology, Institute of Health Carlos III, CIBERESP, Madrid

Clara Mazagatos, National Centre of Epidemiology, Institute of Health Carlos III, CIBERESP, Madrid

Francisco Pozo, National Centre for Microbiology, National Influenza Reference Laboratory, WHO-National Influenza Centre, Institute of Health Carlos III, CIBERESP, Madrid

Inmaculada Casas, National Centre for Microbiology, National Influenza Reference Laboratory, WHO-National Influenza Centre, Institute of Health Carlos III, Madrid

EU-PC study (the cycEVA work group):

Luis García Comas, Dirección General de Salud Pública, Madrid

María Esther Insua Marisquerena, Dirección General de Salud Pública, Madrid

Juan Carlos Galán, Laboratorio Hospital Ramón y Cajal, CIBERESP, Madrid

Mª Dolores Folgueira, Laboratorio Hospital Doce de Octubre, Madrid

Carmen Quiñones Rubio, Dirección General de Salud Pública, Consumo y Cuidados de La Rioja, Logroño

Eva Martinez Ochoa, Dirección General de Salud Pública, Consumo y Cuidados de La Rioja, Logroño

Miriam Blasco, Laboratorio Hospital San Pedro de Logroño, Logroño

Jaume Gimenez Duran, Servicio de Epidemiología, Dirección General de Salud Pública, Mallorca

Juana Maria Vanrell, Servicio de Epidemiología, Dirección General de Salud Pública, Mallorca

Jordi Reina, Laboratorio del Hospital Son Espases, Mallorca

Daniel Castrillejo, Servicio de Epidemiología. DGSC, Consejería de Bienestar Social y Sanidad, Ciudad Autónoma de Melilla, Melilla

ES-PC study (the Spanish Influenza Sentinel Surveillance System):

Concha Delgado, National Centre of Epidemiology, Institute of Health Carlos III, CIBERESP

Jesus Oliva, National Centre of Epidemiology, Institute of Health Carlos III, CIBERESP

EU-H study:

Miriam Latorre-Millán, Instituto de Investigación Sanitaria (IIS) Aragón, Hospital Universitario Miguel Servet, Zaragoza

Ana María Milagro, IIS Aragón, Hospital Universitario Miguel Servet, Zaragoza

Antonio Rezusta, IIS Aragón, Hospital Universitario Miguel Servet, Zaragoza

M. Amelia Fernández Sierra, Medecina Preventiva, Hospital Universitario Virgen de las Nieves, Granada

Mercedes Pérez Ruiz, Hospital Universitario Virgen de las Nieves, Granada

María del Mar Rodríguez, Hospital Universitario Virgen de las Nieves, Granada

Spain – Navarra

EU-PC and EU-H studies:

Jesús Castilla, Instituto de Salud Pública de Navarra – IdiSNA, CIBERESP, Pamplona

Manuel García Cenoz, Navarra Instituto de Salud Pública de Navarra – IdiSNA, CIBERESP, Pamplona

Itziar Casado, Instituto de Salud Pública de Navarra – IdiSNA, CIBERESP, Pamplona

Iván Martínez-Baz, Instituto de Salud Pública de Navarra – IdiSNA, CIBERESP, Pamplona

Carlos Gómez Ibañez, Instituto de Salud Pública de Navarra, Pamplona

Nerea Iriarte, Instituto de Salud Pública de Navarra, Pamplona

Ana Navascués, Complejo Hospitalario de Navarra – IdiSNA, Pamplona

M Eugenia Portillo, Complejo Hospitalario de Navarra – IdiSNA, Pamplona

Carmen Ezpeleta, Complejo Hospitalario de Navarra – IdiSNA, Pamplona

Sweden

EU-PC study:

Mia Brytting, The Public Health Agency of Sweden, Stockholm

Åsa Wiman, The Public Health Agency of Sweden, Stockholm

Theresa Enkirch, The Public Health Agency of Sweden, Stockholm

United Kingdom

UK-PC study:

Jim McMenamin, Health Protection Scotland, Glasgow

Diogo F.P. Marques, Health Protection Scotland, Glasgow

Rory Gunson, West of Scotland Specialist Virology Centre, Glasgow

Chris Robertson, University of Strathclyde, Glasgow

Simon Cottrell, Public Health Wales, Cardiff

Catherine Moore, Public Health Wales, Cardiff

Mark O’Doherty, Public Health Agency Northern Ireland, Belfast

Jillian Johnston, Public Health Agency Northern Ireland, Belfast

Conall McCaughey, Regional Virology Laboratory, Belfast

Simon de Lusignan, University of Oxford, Oxford, Royal College of General Practitioners, London

Ivelina Yonova, University of Oxford, Oxford, Royal College of General Practitioners, London

Manasa Tripathy, University of Oxford, Oxford, Royal College of General Practitioners, London

Rachel Byford, University of Oxford, Oxford

Filipa Ferreria, University of Oxford, Oxford

Jamie LopezBernal, Public Health England, London

Richard Pebody, Public Health England, London

Mary Sinnathamby, Public Health England, London

Heather Whitaker, Public Health England, London

Nick Andrews, Public Health England, London

Joanna Ellis, Public Health England, London

Praveen SebastianPillai, Public Health England, London

Maria Zambon, Public Health England, London
